# Fracture Process Analysis and Acoustic Emission Response of Cemented Tailings Backfill with Different Sizes under Uniaxial Compression

**DOI:** 10.3390/ma15228038

**Published:** 2022-11-14

**Authors:** Kui Zhao, Zhouchao Liu, Peng Zeng, Cong Gong, Yanda Li

**Affiliations:** 1 School of Resources and Environmental Engineering, Jiangxi University of Science and Technology, Ganzhou 341000, China; 2 Engineering Research Institute, Jiangxi University of Science and Technology, Ganzhou 341000, China

**Keywords:** size effect, cemented tailings backfill, uniaxial compression, acoustic emission, digital image correlation technology

## Abstract

To investigate the effect of dimensional changes on the mechanical properties of cemented tailings backfill (CTB), uniaxial compression tests are performed on square CTBs of four different sizes. Combining digital image correlation (DIC) and acoustic emission (AE) methods, the fracture process and AE behavior characteristics of backfills with different sizes are analyzed. The results show that as the backfill size increases, its uniaxial compressive strength decreases, and its strength stabilizes gradually when it measures 100 mm. Under uniaxial compression loading, surface cracks on the smaller specimens evolve rapidly and aggressively, with no significant shedding area, whereas the larger specimens show plastic failure. The cracks expand and penetrate gradually, forming a large shedding area. As the specimen size increases, the backwards trend of the peak value of the ringing count relative to the peak value of the stress becomes increasingly evident. Combining the change law between the *r* value and the average frequency centroid, the sudden drop point of *r* value and the lowest value of average frequency centroid can be regarded as the precursor of macroscopic damage.

## 1. Introduction

As a green mining method, cemented tailings backfill (CTB) technology is widely used in various underground mines in the world because of its advantages in controlling ground pressure, reducing both tailing discharge and the risk of dam break. Thus, in underground filling mining, CTB is used as a substitute for artificial pillars or surrounding rock [1], which is key in providing support and bearing. CTB and rock are typical heterogeneous materials; among them, rock materials exhibit a significant size effect [2]. This factor is considered in practical engineering design and theoretical studies of rock constitutive relationships. However, the size effect of CTB has not been widely investigated.

At the present moment, the size effect of many granular materials has been studied widely. For rock materials, many scholars believe that the size effect is due to two factors [3,4,5,6,7,8,9]. First, the heterogeneity of the material. The amount of internal defects (such as microcracks) in the material increases with the specimen size. Therefore, the mechanical properties of larger specimens are unsatisfactory. Second, when the specimen is subjected to axial compression, the two loading surfaces inevitably form a three-dimensional stress zone at the end owing to friction. With the same boundary conditions, the smaller the size, the greater the impact of the end friction, thus, resulting in improvements in its strength and other properties. Renshaw et al. [10,11] experimentally observed that the stress state at the end of the specimen changed under the boundary conditions of different roughness through tests. The size effect of backfills was investigated previously by some researchers [12,13]. Yilmaz et al. [14] investigated the uniaxial compressive strength (UCS), water content, porosity, and other parameters of three different sizes of cylindrical backfills under four different curing conditions, and reported that the difference in the number of micropores in the specimens contributed to the size effect. Xue et al. [15] conducted uniaxial compression tests on different-sized fiber-reinforced backfills and discovered that size was one of the main factors affecting the mechanical properties of the specimens. Cheng et al. [16] conducted uniaxial compression acoustic emission (AE) and ultrasonic velocity tests on backfills with different sizes and discussed the size effect from two aspects: energy and damage. Guo et al. [17] investigated the change law of strength, AE, and resistivity of backfills with different sizes under uniaxial compression, and discovered that defects such as pores, microcracks, and micropores in the backfill contributed primarily to the size effect. They established damage and constitutive equations for backfills of different sizes. The results above show that heterogeneous materials, such as backfills and rocks, demonstrate a clear size effect. 

In addition, as a nondestructive monitoring method, AE can effectively reflect the internal damage evolution of materials. The possibility of using AE to predict the instability and failure of backfills has been investigated extensively [18,19,20,21]. Qi et al. [22] analyzed the AE counts and resistivity change rule of cemented coal gangue backfill under uniaxial compression at three different curing ages, and found that the change characteristics of resistivity can be better explained from the perspective of AE. Ran et al. [23] found that the AE amplitude can reflect the damage evolution process of the backfill in the single-step creep process. Zhou et al. [24] found that each characteristic stress interval of the backfill under uniaxial compression had a good corresponding relationship with the AE characteristics, and proposed an improved damage variable based on the AE energy rate. However, most of the studies above focused on the AE behavior of single-sized backfills, whereas the AE activities of backfills with different sizes were rarely analyzed, particularly in combination with the evolution of the fracture process of the specimens. 

Previous investigations pertaining to the size effect of backfills primarily focus on the description of apparent phenomena, whereas the overall fracture process and internal damage evolution of backfills with different sizes were rarely investigated. In this study, four groups of different-sized cubic backfills were tested for AE under uniaxial compression. The deformation and failure processes of the specimens were monitored and analyzed based on digital image correlation (DIC) to determine the effect of size change on the mechanical properties of the backfill and the evolution of deformation and fracture, as well as to determine the evolution laws of the AE ringing count, *r* value, and average frequency centroid with loading.

## 2. Materials and Methods

### 2.1. Materials

**Figure 1:**
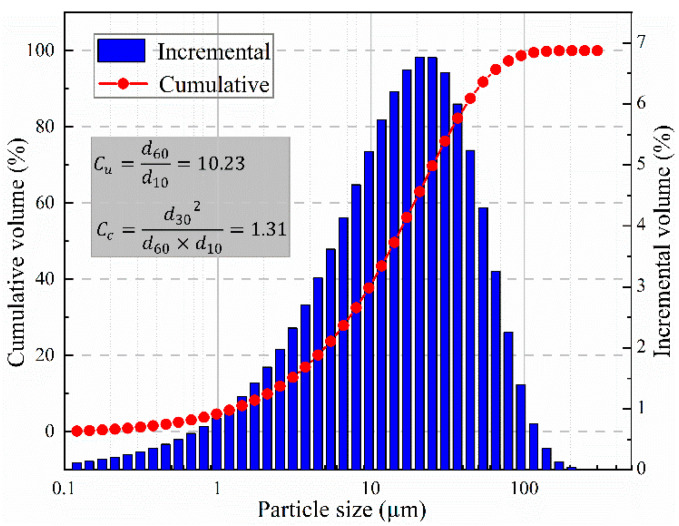
Particle size distribution of tailings.

The tailings used in the specimen preparation were obtained from a copper mine mountain backfill station in Jiangxi Province. The laser particle size analyzer (Winner 2000) was used to detect the particle size of tailings, and the result is shown in Figure 1. The tailings account for approximately 60% of the particles smaller than 20 μm, only approximately 8.5% of the particles are smaller than 2 μm, and approximately 96.7% of the particles are smaller than 75 μm. The particle nonuniformity coefficient and curvature coefficient are 10.23 and 1.31, respectively. In addition, the main chemical components of tailings were determined by X-ray fluorescence spectroscopy (XRD), as shown in Table 1. The cement was P.O 42.5 ordinary composite Portland cement. Ordinary tap water was used for mixing.

### 2.2. Backfill Specimens Preparation

**Figure 2:**
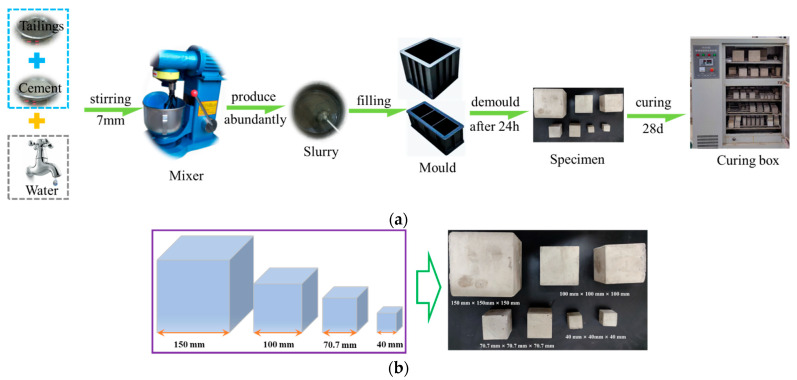
Backfill specimens; (**a**) specimen production process, (**b**) schematic diagram and partial specimens.

The cement-to-tailing ratio was 1:4, and the mass concentration was set to 75%. After mixing the materials homogeneously, the mixture was poured into backfill moulds measuring 40 mm × 40 mm × 40 mm, 70.7 mm × 70.7 mm × 70.7 mm, 100 mm × 100 mm × 100 mm, and 150 mm × 150 mm × 150 mm. After 24 h, it was demolded and placed in a standard curing box to cure for 28 d. The curing temperature was set at (20 ± 2) °C, and the humidity was maintained above 96%. After curing, the size and wave velocity of the specimens were measured, and a backfill with a similar wave velocity was selected for the uniaxial compression test, where three specimens were used for each group. The test specimen preparation process is shown in Figure 2.

### 2.3. Experimental Methods

The uniaxial compression test was performed using the RMT-150C rock mechanics test system developed by the Wuhan Institute of Geotechnical Mechanics, Chinese Academy of Sciences. The loading method used was displacement control, and the loading rate was 0.002 mm/s. In order to ensure the same friction coefficient between the specimen and the loading plate, a polyethylene sheet with the same size as the specimen was placed between the end of the specimen and the loading plate. The AE signal was acquired using the PCI-II AE acquisition system developed and manufactured by the American Physical Acoustics Company. Prior to the test, Vaseline was evenly coated on the surface of the AE probe to ensure complete coupling between the probe and backfill. In addition, to eliminate the impact of environmental noise, the threshold value of AE acquisition system was set to 35 dB. The threshold value of the AE pre-amplifier was set at 40 dB.

**Figure 3:**
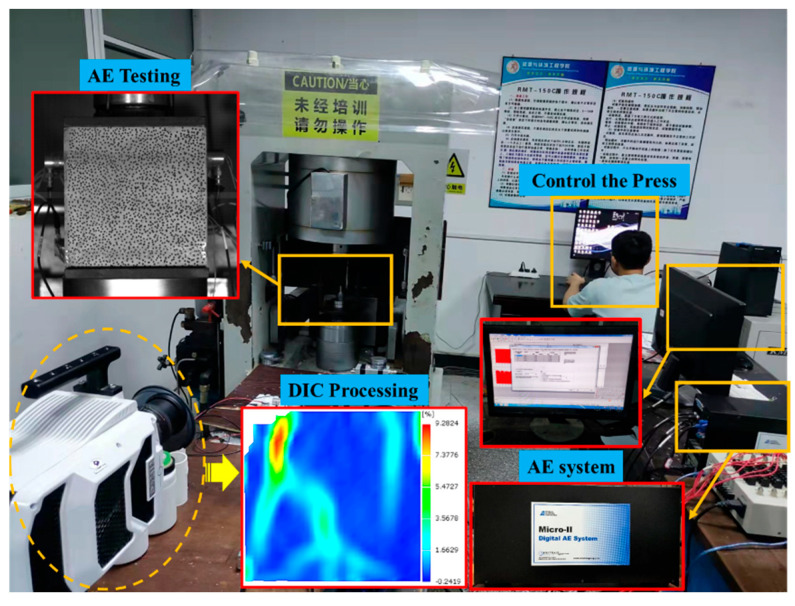
Test equipment.

To investigate the fracture law of different backfill sizes during the loading process, a high-speed camera was used to capture the fracture process of the specimens in real time, and the surface crack evolution law of the specimens based on the uniaxial compression process was determined using the DIC technology. The DIC technology monitors and compares the image pixels of the specimen before and after loading to obtain changes in the surface strain and displacement fields of the specimen. For DIC full-field strain measurements, a layer of white paint was painted on the specimen surface first; subsequently, a random black paint speckle was marked with black markers to obtain a speckle image, after which the DIC technology was applied to monitor and analyze the speckle on the specimen surface before and after loading to obtain the specimen’s surface strain field. The test equipment and speckle specimens are shown in Figure 3.

## 3. Results and Discussion 

### 3.1. Effect of Size Change on UCS and Peak Strain

**Figure 4:**
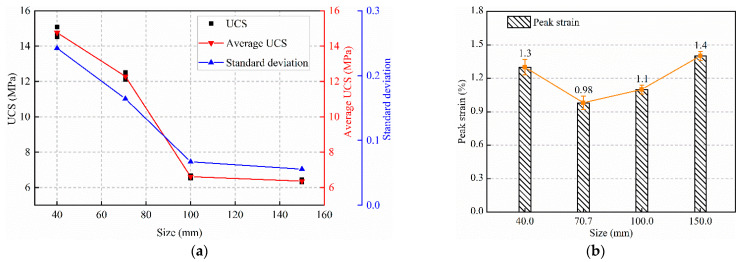
The results of uniaxial compression test; (**a**) UCS curve (**b**) peak strain.

The UCS test values and standard deviation results for backfills with different sizes are shown in Figure 4a. Backfills measuring 40 and 70.7 mm are herein referred to as smaller specimens, whereas those measuring 100 and 150 mm are referred to as larger specimens. Our observations indicate that the backfill size affects its strength. As the size increases, the UCS of the backfill shows a monotonic decreasing trend owing to the heterogeneity of the material. Numerous pores are present in the backfill. A larger backfill can accommodate more pores and is easily damaged by compression. Meanwhile, this leads to a larger friction effect at the end of the smaller specimen, thus, effectively inhibiting crack growth and increasing the strength. It is believed that a primary size exists for rocks of different lithologies, and that the strength of specimens of such a size can be used as the indoor test index [25]. In this study, the primary backfill size is less than 70.7 mm.

Figure 4b shows the relationship between the average peak strain of specimens with different sizes. The peak strain of the specimen measuring 70.7 mm is the lowest. As the size increases, the peak strain of the specimen increases gradually. The peak strain of the specimen measuring 40 mm is between those of the specimens measuring 100 and 150 mm. This indicates that the end effect can result in a higher peak strength in the specimen and increase the peak strain to a certain extent.

Meanwhile, the UCS and standard deviation of the specimen decreases in two stages (see Figure 4a), and the backfills measuring 40 and 70.7 mm show high strength and standard deviation values, indicating that the strength of the smaller specimens is discrete. As the size continues to increase, the reduction range for the standard deviation of the specimen strength decreases, which is consistent with the results of You et al. [3] and Lu et al. [25]. This is because the internal heterogeneity of the specimen decreases gradually with the increase in size. Therefore, the smaller specimens exhibit a significant strength dispersion. When the size reaches a certain critical value, the strength of the specimen stabilizes.

### 3.2. Effect of Size Change on Elastic Modulus and Wave Velocity

**Figure 5:**
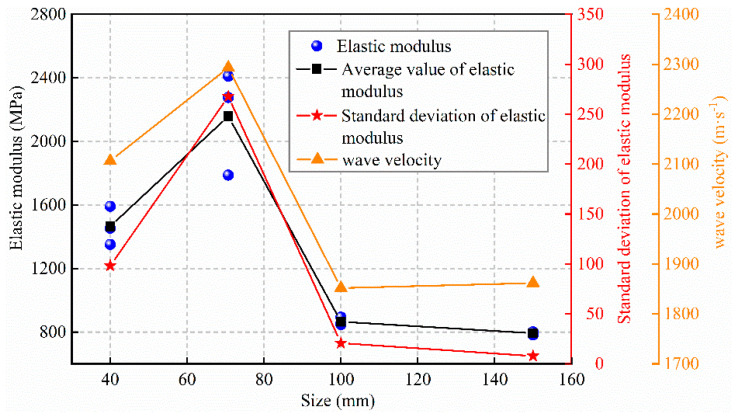
Elastic modulus and wave velocity variation curve of backfills with different sizes.

The slope from the elastic stage of the stress–strain curve of the specimen was selected as the elastic modulus. Figure 5 shows the change curves of the elastic modulus and wave velocity of backfills with different sizes. The wave velocity and elastic modulus trends of the specimen are similar, i.e., both first increase and then decrease. The change in elastic modulus can be predicted using the wave velocity of the specimen. The change in the wave velocity reflects the change in the internal porosity of the specimen [26], i.e., the larger specimens possess higher porosity, whereas the smaller specimens have fewer pores and exhibit higher compactness.

The elastic modulus can be used as an indicator to accurately evaluate the specimen stiffness. Figure 5 shows that the elastic moduli of the 100 and 150 mm specimens are significantly smaller than those of the 40 and 70.7 mm specimens, indicating that the stiffness of the larger specimens is low, which is due to the heterogeneity of the materials. A relatively porous gap is observed in the larger specimens. The elastic moduli of the 40 and 70.7 mm specimens do not match the strength change law. The 40 mm specimen demonstrates higher strength, but its elastic modulus is relatively low. This is because the 40 mm specimen is affected more significantly by the end friction effect. In addition to the axial loading force, its end imposes a lateral friction effect; therefore, it exhibits a longer elastic stage and higher strength. The difference in the elastic modulus between the 100 and 150 mm specimens is insignificant, because as the size increases, the homogeneity difference between specimens decreases gradually, and the size effect weakens gradually [25]. The change trends of the elastic modulus and its standard deviation curve for backfills with different sizes are similar. 

### 3.3. Analysis of Crack Evolution and Stress–Strain Correspondence of Backfills with Different Sizes 

**Figure 6:**
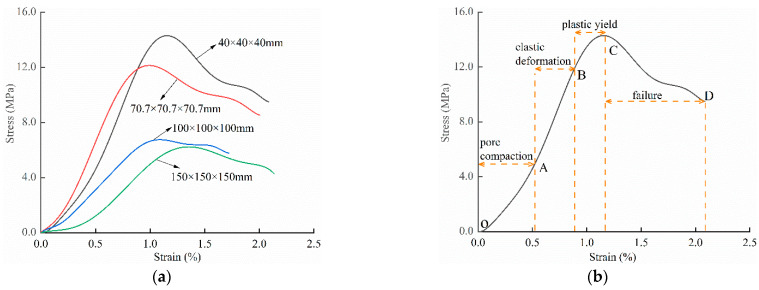
(**a**) Stress–strain curve and (**b**) stage segmentation of backfills with different sizes.

Figure 6a shows the typical stress–strain curves of backfills with different sizes under uniaxial compression. Based on the change law of the curve slope, the stress–strain curve can be segmented into four stages, as shown by the yellow arrows in Figure 6b: pore compaction stage (OA section), linear elasticity stage (AB section), plastic yield stage (BC section), and post-peak failure stage (CD section) [24]. 

Under uniaxial loading, the corresponding relationship between the main strain field evolution nephogram and the stress–strain curve stage of typical backfills of different sizes after DIC treatment is presented in Table 2. A typical main strain field nephogram was selected for each stage; the strain field nephogram obtained after DIC treatment reflected the stress concentration and crack evolution process of the backfills during loading more accurately.

In the initial loading stage, i.e., the pore compaction stage (OA section), the surface strain field of the backfill does not transform significantly, and the entirety is shown in blue. As the loading proceeds and enters the linear elastic stage (AB section), the strain concentration area gradually appears on the specimen surface, as represented by the blue and white areas in the figure, which unfolds in a strip. The strip-shaped strain concentration area continues to extend and expand as the test progresses, forming a strain concentration zone gradually. In the plastic yield stage (BC section), the DIC results show that the stress concentration becomes e increasingly evident, and the yellow and green areas in the corresponding figure expand rapidly, indicating that the microcracks in the backfill are continuously connected to each other and are in the early stage of failure. When the peak stress point (point C) is reached, macroscopic cracks appear on the backfill surface, corresponding to the red strain concentration area, which further expands as loading occurs (CD section). The red area expands, and the backfill specimen enters the post-peak failure stage, which may be accompanied by the surface fall-off of the backfill specimen. The principal range of strain change associated with the specimen failure occurs after the peak stress. 

**Figure 7:**
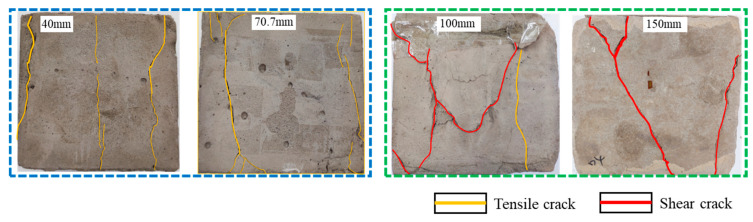
Failure patterns of backfills.

A comparison of the DIC treatment results of backfills with different sizes shows that the strain concentration area of the smaller specimens is significantly larger than that of the larger specimens. For the 40 and 70.7 mm specimens, damage to the backfills is caused by multiple strain concentration zones on the left and right sides, which are affected by the end effect. For the 100 and 150 mm specimens, the damage strain concentration zone of the backfill is concentrated in the middle of the specimens, accompanied by the generation of some small cracks. Meanwhile, the DIC results show that as the specimen size increases, the peak principal strains (corresponding to the principal strains in the red region) of the specimens are 1.51%, 3.89%, 5.68%, and 9.28%, showing an increasing trend. The phenomena show that the smaller specimens demonstrate higher compactness. Under uniaxial compression, they exhibit the characteristics of partial elastic brittle failure. The surface crack evolution is rapid and intense, and a fall-off zone is not indicated. The failure pattern of the specimen is the coexistence of tension failure and shear failure. Meanwhile, the larger specimen exhibits plastic failure, where cracks gradually expand and penetrate. The main failure pattern is shear failure, which results in a large fall-off zone. In Figure 7, the failure images (non-speckled surface) of specimens of all sizes are displayed. It can be seen that the failure surfaces of small size specimens are relatively smooth, with 2–3 main cracks and multiple small cracks, and the failure pattern is dominated by tension action. For large size specimens, the failure is mainly caused by the through main crack, accompanied by a large area of collapse, and the failure pattern is dominated by shear action. Therefore, the results in Figure 7 and Table 2 are mutually verified.

### 3.4. AE Response of Crack Propagation in Backfills with Different Sizes 

During the deformation of the backfills, the internal pores are gradually compacted, and the microcracks gradually expand and connect with each other. Simultaneously, the energy accumulated in the backfill is released in the form of elastic waves. This phenomenon is known as AE [27]. Therefore, we can obtain and analyze the AE information during the loading process of the backfill to investigate the precursor to its failure. 

#### 3.4.1. Crack Fracture Process and Evolution of AE Time Series 

**Figure 8:**
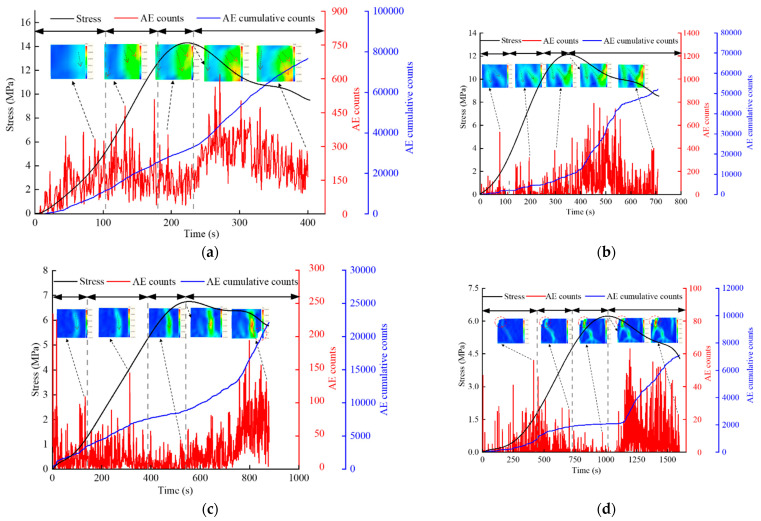
Crack propagation and evolution process and corresponding AE time series response of backfills of different sizes: (**a**) 40 mm × 40 mm × 40 mm; (**b**) 70.7 mm × 70.7 mm × 70.7 mm; (**c**) 100 mm × 100 mm × 100 mm; (**d**) 150 mm × 150 mm × 150 mm.

The characteristics of the deformation field on the backfill surface were obtained using the DIC technology. Figure 8 shows the corresponding relationship between the AE ringing count, evolution nephogram of the main strain field, and stage of the stress–time curve of four typical backfills of different sizes. 

In general, the peak value of the ringing count of the backfill appears after the peak stress value, and the ratio of relative lag time to the corresponding time of peak stress is considered as the relative lag rate. The calculation results are presented in Table 3. As the size increases, the backwards trend of the peak value of the ringing count relative to the peak value of the stress becomes increasingly evident, which corresponds to the significant change range of the specimen damage in the DIC analysis results. The AE ringing count and corresponding crack evolution characteristics at each stress stage of the specimens with different sizes are described as follows: 

In the initial compaction stage, the specimens of four different sizes demonstrate clear AE; however, the specimen surface does not show significant cracks, which are primarily caused by the compact pores in the backfill. The continuity of the AE ringing count decreases as the size increases. At the beginning of the loading process, the larger specimen releases a large AE signal. This is because the larger specimen contains more large pores, and the internal cracks are easily compressed and closed. However, the smaller specimen is significantly affected by the end “clamping” effect at this time, and the pore compaction is limited [9]. In the online elastic stage, the surface strain concentration of the specimen is more significant than that in the other stages. The AE phenomenon is primarily due to the friction between the pores and microcracks in the backfill under axial loading. The cumulative ringing count curve of the four specimen sizes increases gradually. In the plastic yield stage, the strain concentration area on the surface of the specimen continues to expand, the AE ringing count of the backfill is fewer, and a relatively “quiet period” occurs. This is because the backfill contains more pores and microcracks, the pores and other defects are easily destroyed under uniaxial compression, and the strength of the cement bond is higher, which results in “cracking without breaking.” In the post-peak stage, the internal microcracks of the specimen gradually expand under the load, the friction between particles on the fracture surface of the backfill is high, and the AE ringing count continues to increase. At the peak stress, a high strain concentration area appears on the surface of the specimen, indicating the formation of a macrofracture surface. Additionally, the microcracks penetrate the surface, resulting in internal damage to the backfill. At this time, the AE ringing count increases significantly.

#### 3.4.2. Joint Analysis of AE *r* Value and Frequency-Domain Characteristics 

The release law of AE energy can effectively reflect the scale of crack derivation in the backfill. To investigate the energy change law of AE signals generated during specimen loading in the time- and frequency-domains, the *r* value and average frequency centroid of the AE in the backfill were analyzed statistically.

The *r* value is defined as the ratio of cumulative AE events to the cumulative energy, i.e., r=∑N/∑E. Based on this definition, the *r* value increases as the number of AE events increases and the energy decreases, whereas it decreases as the number of events decreases and the energy increases. Therefore, some scholars [28,29,30,31,32] believe that an increase in *r* represents the closure of microcracks in the specimen and friction between particles, which results in more low-energy AE signals. Meanwhile, a decrease in *r* represents the mutual penetration of microcracks and the gradual formation of macrocracks, which is accompanied by the occurrence of high-energy AE events. The frequency centroid is calculated by dividing the sum of the AE amplitude spectrum and its frequency product by the sum of the amplitude spectrum. This change can reflect the distribution characteristics of the AE energy in the frequency domain. That is, the higher the energy of the AE high-frequency signal, the larger the frequency centroid, and vice versa. In this study, the average value of the frequency centroid of the AE signal generated by the specimen per second, which is known as the average frequency centroid, was obtained [33].

**Figure 9:**
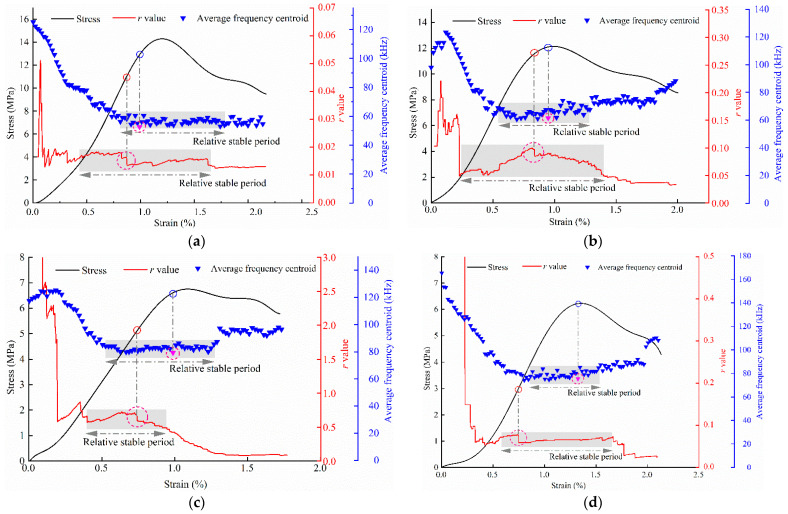
Relationship among AE *r* value, and stress and strain of backfills with different sizes; (**a**) 40 mm × 40 mm × 40 mm, (**b**) 70.7 mm × 70.7 mm × 70.7 mm, (**c**) 100 mm × 100 mm × 100 mm, (**d**) 150 mm × 150 mm × 150 mm.

Figure 9 shows the relationship among the *r* value, average frequency centroid, and stress–strain curve of backfills with different sizes. The *r* value and average frequency centroid of the backfills with different sizes exhibit a similar change trend with loading, i.e., it first increases rapidly to a higher value and then oscillates to a relatively stable level. At a certain point, the *r* value decreases abruptly to a relatively stable value, as shown by the pink circle in Figure 9. The abrupt change indicates an increase in high-energy events and represents intense crack propagation in the specimen. The average frequency centroid decreases gradually in the relatively stable stage and increases after reaching a minimum value near the peak stress, as shown by the pink circle in Figure 7. Combining the change law of the *r* value, the lowest value of the average frequency centroid indicates that the high-frequency and high-energy events of the specimen AE signal decrease gradually, whereas the low-frequency and high-energy events increase. The sudden drop point of *r* value and the lowest value of average frequency centroid can be regarded as the precursor of macroscopic damage.

As shown in Figure 9, the *r* values of the 40 and 70.7 mm backfills change significantly during the loading process, whereas those of the 100 and 150 mm backfills fluctuate significantly only in the initial loading stage, after which they decrease rapidly and stabilize. As the size increases, the *r* value corresponding to the descending mutation point of the specimen indicates an overall increasing trend, whereas the descending mutation amplitude of the *r* value indicates a decreasing trend. The descending amplitudes of the 40, 70.7, 100, and 150 mm specimen are 24.58%, 14.74%, 13.17%, and 12.51%, respectively. This indicates that the smaller specimens generate more high-energy events during the entire uniaxial loading process, which corresponds to more intense interactions among the internal particles. Meanwhile, the larger specimens indicate low compactness and energy storage capacity, and its loading process is primarily accompanied by low-energy events. Therefore, the smaller specimens exhibit brittle elastic failure characteristics, whereas the larger specimens primarily exhibit plastic failure.

### 3.5. Cause Analysis of Size Effect 

**Figure 10:**
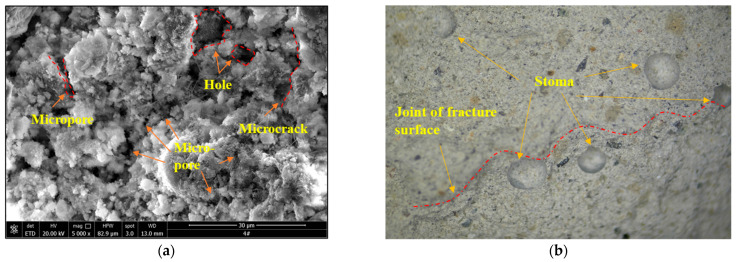
Microstructure of damaged backfill; (**a**) optical microscopy, (**b**) scanning electron microscopy.

To further investigate the factor contributing to the size effect of the backfill, the microstructure of the damaged backfill was analyzed using a polarizing microscope and a scanning electron microscope, as shown in Figure 10. The analysis shows that the backfill is a typical porous heterogeneous material. The products generated by hydration, such as ettringite, hydrated calcium silicate gel, and calcium hydroxide, are distributed in a disordered manner. During the hydration process, numerous pores are generated easily, and the hydration products cannot completely encapsulate the sand particles at the end of the glue. Many defects are observed in the backfill, such as pores, micropores, and microcracks.

The size effect is the average effect of multiple factors. The existence of various defects in the backfill and their disordered development contribute to the size effect of the backfill via two aspects: (1) The internal cementation strength of the backfill is low, and the defect structure contained in the backfill is easily damaged and connected under load. Meanwhile, a larger size implies more defects in the backfill; thus, the overall bearing capacity and energy storage capacity of the larger backfill are reduced and the strength is reduced. (2) The type and size of the defects are disordered. The smaller the size of the specimen, the greater the effect of the random distribution (heterogeneity) of the defects. The larger the specimen size, the higher the tolerance of the random distribution of defects, which is reflected in the higher dispersion of the strength and elastic modulus in the smaller specimens.

**Figure 11:**
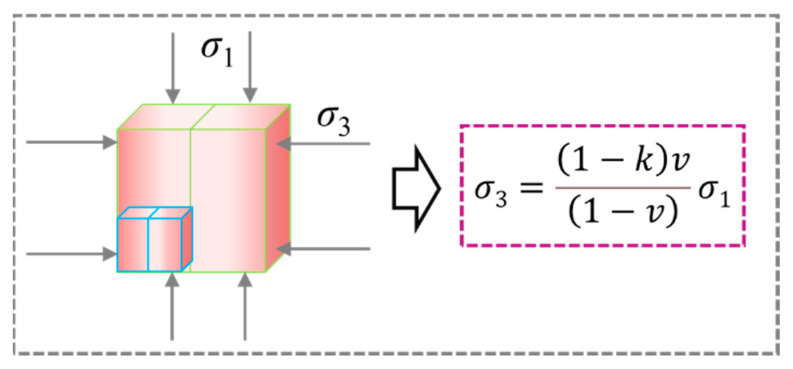
Schematic diagram of friction effect at the end of backfills.

**Figure 12:**
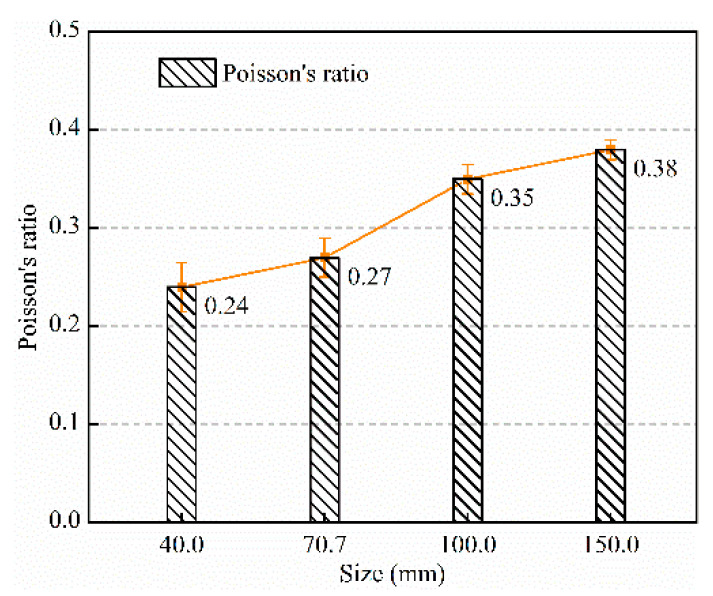
Poisson’s ratio of backfills with different sizes.

Combined with Figure 4 and Figure 5, it can be found that with the size change, the elastic modulus of the specimen presents a different change law from the strength, the strength of the specimen decreases monotonically with the size increase, and the elastic modulus reaches the maximum when the size is 100 mm. Similar problems also exist in rocks. Yang et al. [5,6] explained this situation. On the one hand, the existence of defects reduces the strength and elastic modulus of specimens with the increase in size. On the other hand, due to the end friction, the end of the specimen is “clamped”, forming a triaxial compression zone, as shown in Figure 11. According to the generalized Hooke’s law, Guo [34] gave the relationship between the lateral restraint stress on the end of the rock specimen (*σ*_3_) and axial stress (*σ*_1_), where *υ* is Poisson’s ratio, *k* is friction correlation coefficient, 0 < *k* < 1. It can be found that when the specimen is subjected to the same axial stress and has the same friction correlation coefficient, the lateral restraint stress increases with increasing Poisson’s ratio. Poisson’s ratio of each size specimen under uniaxial compression test is shown in Figure 12, indicating that the larger size specimen has a larger Poisson’s ratio. Thus, in the initial loading stage, when the specimen is subjected to the same axial stress, the larger the size of the specimen, the stronger the end restraint force. However, due to the existence of defects, the smaller the size of the specimen, the greater the axial stress it can bear, so the end restraint stress caused by the friction effect is stronger, which may be the main reason for the results of this study. In the smaller size and lower stress range, the end effect plays a major role, thus, increasing the elastic modulus of the specimen. When the size exceeds this range, the effect of the defect is greater than the end effect, and the elastic modulus decreases with the increase in the size. In conclusion, end friction and defects are the main reasons contributing to the size effect of the backfill. 

## 4. Conclusions

Based on the uniaxial compression AE tests of backfills with different sizes, the effect of size change on the strength characteristics, elastic modulus, stress–strain curve, and AE characteristics of backfills was discussed. Additionally, the failure process of backfills with different sizes based on the DIC technology was analyzed. The main conclusions are as follows:

(1) The backfill demonstrates a clear size effect: as the size increases, the UCS of the specimen decreases in two stages, and the strength stabilizes after the size reaches 100 mm. The elastic modulus and initial wave velocity trends are similar. The elastic modulus and wave velocity of the smaller specimens are significantly greater than those of the larger specimens. The end friction effect results in a higher peak strength and increases the peak strain;
(2) The surface cracks of the smaller specimens evolve rapidly and aggressively without a fall-off zone under uniaxial compression. Meanwhile, the larger specimens are plastically damaged in general, where cracks gradually expand and penetrate, which facilitates the formation of a large fall-off zone;
(3) As the specimen size increases, the backwards trend of the peak value of the ringing count relative to the peak value of the stress becomes increasingly evident. The occurrence of a change point at which the AE *r* value decreases abruptly indicates an increase in high-energy events, whereas the lowest average frequency centroid indicates a decrease in high-frequency high-energy events and an increase in low-frequency high-energy events. The sudden drop point of *r* value and the lowest value of average frequency centroid can be regarded as the precursor of macroscopic damage.

## Figures and Tables

**Table 1:** The main chemical components of tailings.

Component	SiO_2_	CaO	Al_2_O_3_	K_2_O	TiO_2_	P_2_O_5_
Content/%	71.56	1.12	11.62	0.51	0.19	0.13

**Table 2:** Nephogram of strain field evolution of backfills with different sizes.

Size (mm)	OA	AB	BC	C	CD
40	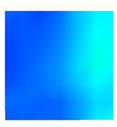	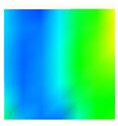	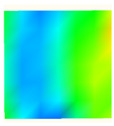	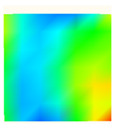	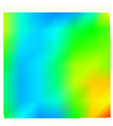
	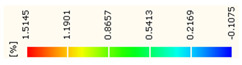				
70.7	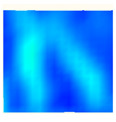	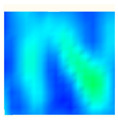	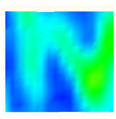	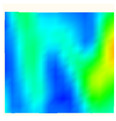	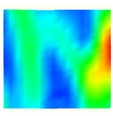
	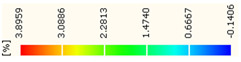				
100	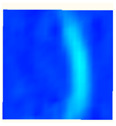	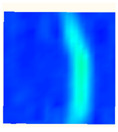	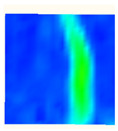	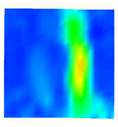	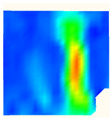
	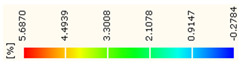				
150	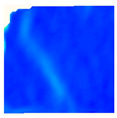	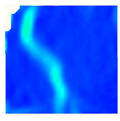	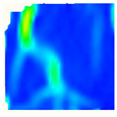	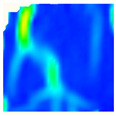	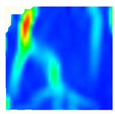
	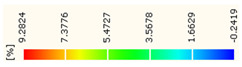				

**Table 3:** Ringing count peak relative stress peak lag result.

Size (mm)	*T*_1_ (s)	*T*_2_ (s)	Δ*T* (s)	Relative Lag Ratio
40	243	262	19	0.078
70.7	358	438	80	0.223
100	524	791	267	0.510
150	1104	1370	230	0.208

Notes: *T*_1_ is the corresponding time of peak stress; *T*_2_ is the corresponding time of the peak ringing count; Δ*T* is the relative lag time.

## Data Availability

Not applicable.
